# P-1294. Epidemiology of Carbapenem-resistance Mechanisms and the Utility of Newly Introduced Beta-lactams/beta-lactam Inhibitors in Patients with Pseudomonas aeruginosa Bacteremia

**DOI:** 10.1093/ofid/ofaf695.1482

**Published:** 2026-01-11

**Authors:** Young Kyung Yoon, Yoon Hyun Sung, Jeong Yeon Kim, Jang Wook Sohn

**Affiliations:** Korea University College of Medicine, Seoul, Seoul-t'ukpyolsi, Republic of Korea; Korea University College of Medicine, Seoul, Seoul-t'ukpyolsi, Republic of Korea; Korea University College of Medicine, Seoul, Seoul-t'ukpyolsi, Republic of Korea; Korea University College of Medicine, Seoul, Seoul-t'ukpyolsi, Republic of Korea

## Abstract

**Background:**

The aim of this study was to analyze the molecular epidemiology of carbapenem-resistance in blood isolates of carbapenem-resistant *Pseudomonas aeruginosa* (CRPA) and the cross-resistance to new beta-lactams/beta-lactam inhibitors.

**Methods:**

In a prospective observational study, CRPA isolates were collected from patients diagnosed with CRPA bacteremia in a university-affiliated hospital of the Republic of Korea (ROK) from May 2020 to February 2025. CRPA isolates were tested for antibiotic susceptibility to ceftolozane-tazobactam (C/T), ceftazidime-avibactam (CZA), imipenem-relebactam (I/R), meropenem-vaborbactam (MVB), and aztreonam-avibactam (AZA) using the broth microdilution method. Molecular mechanisms of carbapenem-resistance were determined by polymerase chain reaction (PCR) for six resistance genes (*bla*_VIM_, *bla*_IMP_, *bla*_NDM_, *bla*_GES_, *bla*_KPC_, and *bla*_OXA-48_). Real-time reverse transcription PCR analysis was performed to confirm reduced expression of *oprD* or overexpression of *mexB, mexD, mexF, mexY*, and *ampC*.

**Results:**

A total of 51 patients with CRPA bacteremia were included in our analysis. In-hospital mortality rate was 52.9%. The MIC_50_/MIC_90_ of the CRPA isolates of C/T, CZA, I/R, MVB, and AZA were 4/ >256 mg/L, 16/ >256 mg/L, 8/ >256 mg/L, 32/ >256 mg/L, and 8/16 mg/L, respectively. AZA showed the highest susceptibility (90.20%), followed by C/T (50.98%), CZA (41.18%), I/R (23.53%), and MVB (19.61%). The proportion of isolates producing carbapenememase was 45.1%. Among them, *bla*_NDM_ was the most common at 82.6%, followed by *bla*_IMP_ (26.1%) and *bla*_GES_ (17.4%). Among all isolates, porin loss and efflux pump overexpression were confirmed in 80.4% and 27.5%, respectively. Isolates non-susceptible to C/T showed 4.0% and 92.0% susceptibility to CZA and AZA, respectively, and resistance to both I/R and MVB. On the other hand, isolates non-susceptible to CZA showed 20.0% and 93.3% susceptibility to C/T and AZA, respectively, and resistance to both I/R and MVB.

**Conclusion:**

In the ROK, CRPA isolates showed high resistance rates to C/T and CZA at 45.1% and 58.8%, respectively. Meanwhile, they showed the highest susceptibility at 90.2% to AZA. Therefore, the introduction of new antibiotics such as AZA is required to overcome the carbapenem-resistance of CRPA isolates in the ROK.

**Disclosures:**

All Authors: No reported disclosures

